# Peiminine Protects against Lipopolysaccharide-Induced Mastitis by Inhibiting the AKT/NF-κB, ERK1/2 and p38 Signaling Pathways

**DOI:** 10.3390/ijms19092637

**Published:** 2018-09-06

**Authors:** Qian Gong, Yanwei Li, He Ma, Wenjin Guo, Xingchi Kan, Dianwen Xu, Juxiong Liu, Shoupeng Fu

**Affiliations:** College of Veterinary Medicine, Jilin University, Changchun 130062, China; qianqiangong2018@163.com (Q.G.); ywli17@mails.jlu.edu.cn (Y.L.); mahe9916@mails.jlu.edu.cn (H.M.); guowenjin2008@126.com (W.G.); kanxc16@mails.jlu.edu.cn (X.K.); xudw9913@mails.jlu.edu.cn (D.X.)

**Keywords:** peiminine, mastitis, LPS, AKT/NF-κB, ERK1/2, p38

## Abstract

Peiminine, an alkaloid extracted from Fritillaria plants, has been reported to have potent anti-inflammatory properties. However, the anti-inflammatory effect of peiminine on a mouse lipopolysaccharide (LPS)-induced mastitis model remains to be elucidated. The purpose of this experiment was to investigate the effect of peiminine on LPS-induced mastitis in mice. LPS was injected through the canals of the mammary gland to generate the mouse LPS-induced mastitis model. Peiminine was administered intraperitoneally 1 h before and 12 h after the LPS injection. In vitro, mouse mammary epithelial cells (mMECs) were pretreated with different concentrations of peiminine for 1 h and were then stimulated with LPS. The mechanism of peiminine on mastitis was studied by hematoxylin-eosin staining (H&E) staining, western blotting, and enzyme-linked immunosorbent assay (ELISA). The results showed that peiminine significantly decreased the histopathological impairment of the mammary gland in vivo and reduced the production of pro-inflammatory mediators in vivo and in vitro. Furthermore, peiminine inhibited the phosphorylation of the protein kinase B (AKT)/ nuclear factor-κB (NF-κB), extracellular regulated protein kinase (ERK1/2), and p38 signaling pathways both in vivo and in vitro. All the results suggested that peiminine exerted potent anti-inflammatory effects on LPS-induced mastitis in mice. Therefore, peiminine might be a potential therapeutic agent for mastitis.

## 1. Introduction

It is well known that dairy mastitis is a production-limiting disease that is harmful to the dairy industry. The causes of mastitis are different, mainly due to the invasion of bacteria, fungi, and pathogens into mammary epithelial cells. Among them, *Escherichia coli* is the most common pathogenic bacteria [1], and lipopolysaccharide (LPS) from gram-negative bacteria is considered an important factor that is used to establish an animal model of inflammation [2]. LPS induces a strong inflammatory response, through the production of pro-inflammatory mediators, such as tumor necrosis factor alpha (TNF-α), interleukin-6 (IL-6), and interleukin-1β (IL-1β) [3,4], and the genes of these pro-inflammatory factors are regulated by the nuclear factor-κB (NF-κB), ERK1/2, and p38 signaling pathways [5]. In clinical practice, the use of large amounts of antibiotics leads to serious drug resistance, a decreased efficacy, and the emergence of superbugs. However, the advantages of natural Chinese herbal extracts, including little side effects, light damage to substantial organs, and a good effect on inflammation, are increasingly favored by researchers.

**Figure 1:**
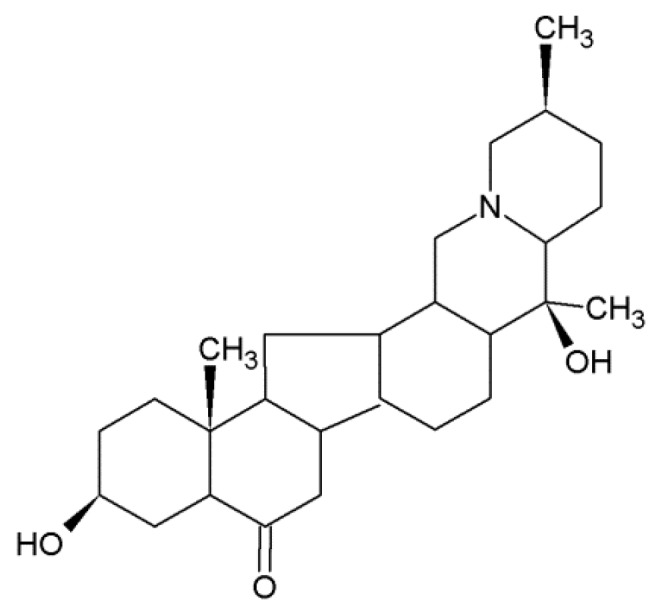
Chemical structure of peiminine.

More and more studies focus on natural products and mastitis. Previous studies have shown that mangiferin can inhibit mastitis induced by LPS via suppressing NF-κB and NLRP3 signaling pathways, and resveratrol inhibits LPS-induced mice mastitis through attenuating the Mitogen-activated protein kinase (MAPK) and NF-κB signaling pathway [6,7]. Peiminine is a natural product of alkaloids, which comes from Fritillaria [8]. Peiminine (Figure 1) is a monomer derived from a traditional Chinese medicinal herb. Studies show that peiminine represses colorectal carcinoma tumor growth by inducing autophagic cell death [9]. Peiminine also plays a role in alleviating bleomycin-induced acute lung injury in rats [10]. In addition, studies demonstrate that peiminine has good anti-inflammatory, antitussive, and expectorant effects [11]. Although peiminine shows good therapeutic effects on other diseases, its role and mechanism in protection against mastitis are not clear. The purpose of this study was to investigate the anti-inflammatory effect and mechanism of peiminine on a mouse LPS-induced mastitis model.

## 2. Results

### 2.1. Effects of Peiminine on the LPS-Induced Histopathological Impairment of the Mammary Gland

**Figure 2:**
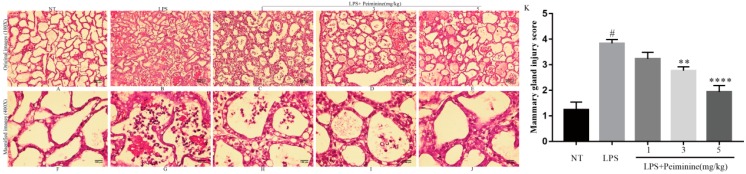
Histopathological sections of the mammary tissues (H&E, 100× and 400×). The mammary tissues (*n* = 6 in each group) from each group were processed for histological evaluation. Representative histological changes in the mammary tissues from each group were as follows: (**A**,**F**) no-treatment (NT) group; (**B**,**G**) lipopolysaccharide (LPS) group; (**C**,**H**) LPS + 1 mg/kg peiminine group; (**D**,**I**) LPS + 3 mg/kg peiminine group; and (**E**,**J**) LPS + 5 mg/kg peiminine group. Histologic grade of the mammary gland (**K**). The histological morphology and pathology results showed that peiminine treatment alleviated the LPS-induced pathological changes. The number sign (#) indicates a significant difference from the NT group at *p* < 0.0001. The tetrad asterisks (****) indicate *p* < 0.0001 and the double asterisks (**) indicate *p* < 0.01 vs. the LPS group.

The histopathological changes are shown in Figure 2A–J. Overall mammary gland injury was scored based on edema, neutrophil infiltration, and hemorrhage, and three visual fields were observed for each slice. Studies were performed in a blinded manner. Injury scores were representative of severity (0, no damage; 1, mild damage; 2, moderate damage; 3, severe damage; 4, very severe damage) (Figure 2K). As shown in Figure 2, the no-treatment (NT) group displayed no abnormal histopathological changes (Figure 2A,F). After LPS treatment, the structure of the mammary gland vesicles was damaged, the wall of the gland was thickened, and the mammary gland contained extensive inflammatory cell infiltration (Figure 2B,G). However, compared with the LPS group, the peiminine treatment groups displayed reduced cellular infiltration and decreased abnormal histopathological changes in a dose-dependent manner (Figure 2C–E,H–J). Importantly, the group treated with 5 mg/kg peiminine was nearly identical to the NT group (Figure 2E,J).

### 2.2. Effect of Peiminine on Myeloperoxidase (MPO) Activity and Pro-Inflammatory Mediators in the Mammary Gland of LPS-Induced Mastitis Model Mice

**Figure 3:**
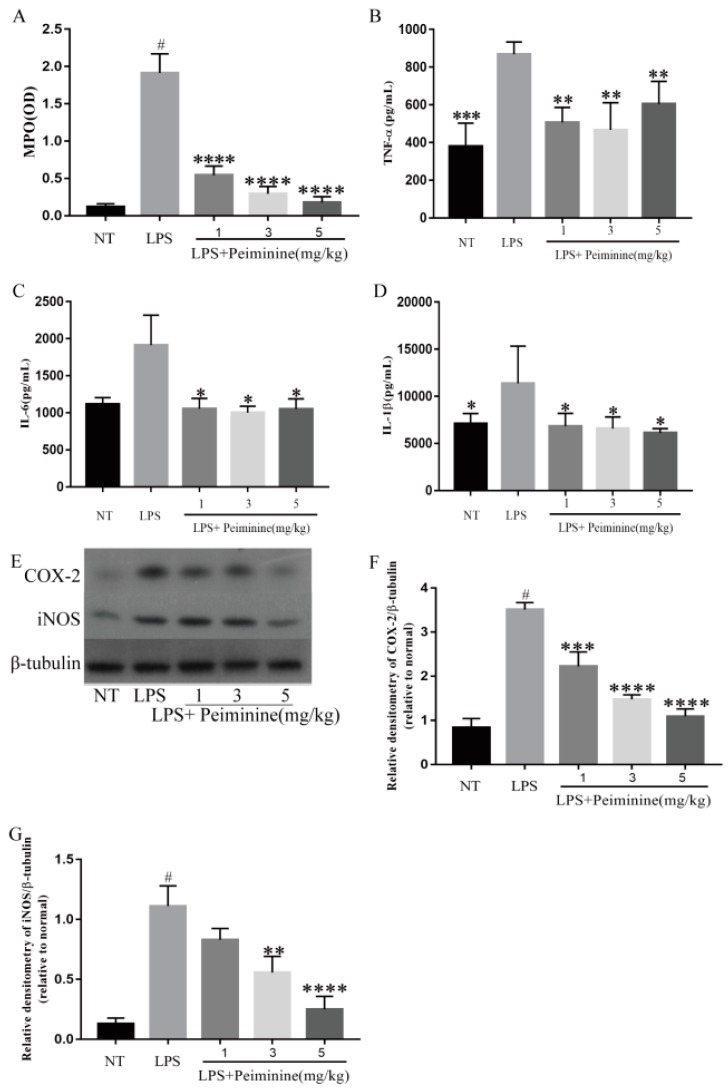
Effect of peiminine on (**A**) the myeloperoxidase (MPO) activity in the mammary gland of LPS-induced mastitis, the levels of (**B**) tumor necrosis factor alpha (TNF-α), (**C**) interleukin-6 (IL-6), and (**D**) interleukin-1β (IL-1β) in the homogenate of the mouse mammary tissues, including the NT group, the LPS group, and the groups treated with peiminine (1, 3, and 5 mg/kg), and the protein levels of (**E**,**F**) COX-2 and (**E**,**G**) iNOS were measured by western blotting. The data are presented as the mean ± SD (*n* = 6). The number sign (#) indicates *p* < 0.0001, which was significantly different from the NT group. The tetrad asterisks (****) indicate *p* < 0.0001, the triple asterisks (***) indicate *p* < 0.0005, the double asterisks (**) indicate *p* < 0.01, and the single asterisk (*) indicates *p* < 0.05 vs. the LPS group.

Compared with the NT group, the LPS-treated group had significantly increased MPO activity in the mammary tissues (*p* < 0.0001) (Figure 3A). In addition, peiminine significantly reduced the MPO activity in mice with LPS-induced mastitis (*p* < 0.0001) (Figure 3A). The pro-inflammatory mediators TNF-α, IL-6, IL-1β, cyclooxygense-2 (COX-2), and induced nitric oxide synthase (iNOS) play very important roles in the process of inflammation [12]. The production of TNF-α (Figure 3B), IL-6 (Figure 3C), and IL-1β (Figure 3D) in the mammary tissues was detected by enzyme-linked immunosorbent assay (ELISA), and COX-2 (Figure 3E,F) and iNOS (Figure 3E,G) levels were detected by western blotting. The results showed that compared with the NT group, the LPS-treated groups had significantly increased production of TNF-α, IL-6, IL-1β, COX-2, and iNOS, and that peiminine inhibited the production of these pro-inflammatory mediators (Figure 3B–G).

### 2.3. Effect of Peiminine on the LPS-Induced Activation of AKT, NF-κB p65, ERK1/2, and p38 in Mammary Tissues

**Figure 4:**
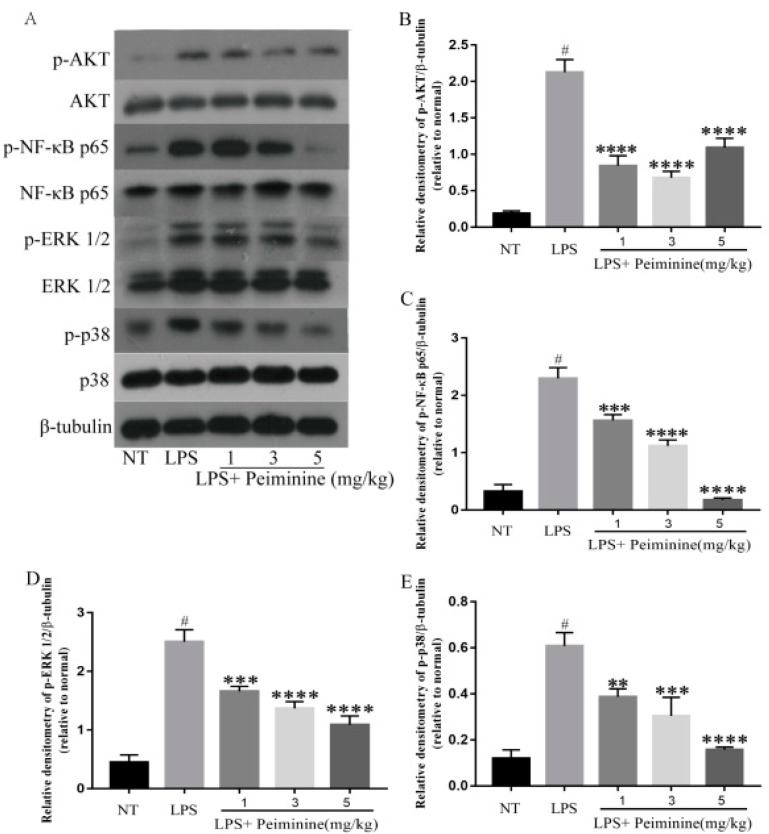
Peiminine inhibits the phosphorylation of the AKT, nuclear factor-κB (NF-κB) p65, ERK1/2 and p38 signaling pathways in the mammary tissues. The results for the protein levels of (**A**,**B**) p-AKT, (**A**,**C**) p-NF-κB p65, (**A**,**D**) p-ERK1/2, and (**A**,**E**) p-p38 were measured by western blotting. The data are presented as the mean ± SD (*n* = 6). The number sign (#) indicates a significant difference from the NT group at *p* < 0.0001. The tetrad asterisks (****) indicate *p* < 0.0001, the triple asterisks (***) indicate *p* < 0.0005, and the double asterisks (**) indicate *p* < 0.01 vs. the LPS group.

The NF-κB signaling pathway, which is activated by toll-like receptors (TLRs), plays a pivotal role in regulating the expression of pro-inflammatory mediators. To explore how peiminine modulated the inflammatory response in the mammary tissues, the phosphorylation of NF-κB and its upstream kinase AKT were measured by western blotting. The results showed that peiminine significantly suppressed the LPS-induced phosphorylation of NF-κB p65 and AKT in a dose-dependent manner (Figure 4A–C). The ERK1/2 and p38 signaling pathways are also important in the inflammatory response. Thus, the effect of peiminine on the activation of ERK1/2 and p38 was examined. LPS significantly increased the phosphorylation of ERK1/2 and p38 (Figure 4A,D,E). As expected, in the presence of peiminine, the increased phosphorylation of ERK1/2 and p38 upon LPS stimulation was attenuated (Figure 4A,D,E).

### 2.4. Effect of Peiminine on Cell Viability and LPS-Induced Inflammatory Response in mMECs

**Figure 5:**
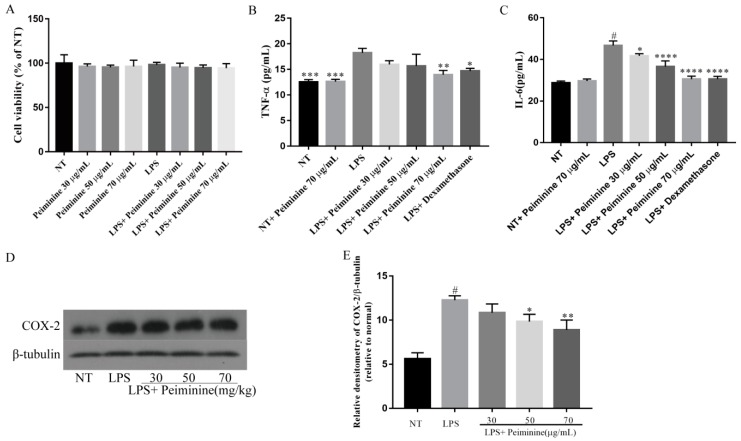
Effect of peiminine on (**A**) cell viability. The cells were cultured with different concentrations of peiminine (30, 50, or 70 μg/mL) for 4 h. Cell viability was determined by the CCK8 assay. The levels of (**B**) TNF-α and (**C**) IL-6 in the mouse mammary epithelial cells (mMECs), including the NT group, the NT + peiminine 70 μg/mL group, the LPS group, and the groups treated with peiminine (30, 50, or 70 μg/mL), and the protein level of (**D**,**E**) COX-2 were measured by western blotting. The data are presented as the mean ± SD (*n* = 6). The number sign (#) indicates a significant difference from the NT group at *p* < 0.0001. The tetrad asterisks (****) indicate *p* < 0.0001, the triple asterisks (***) indicate *p* < 0.0005, the double asterisks (**) indicate *p* < 0.01, and the single asterisk (*) indicates *p* < 0.05 vs. the LPS group.

To examine the cytotoxicity of peiminine, the mouse mammary epithelial cells (mMECs) were treated with peiminine at concentrations ranging from 30 to 70 μg/mL for 1 h and were then stimulated with LPS for 4 h. Then, the cell viability was detected by a cell counting kit-8 (CCK8) assay. The CCK8 results showed that peiminine did not affect cell viability (Figure 5A). The production of TNF-α and IL-6 in the mMECs was detected by ELISA, and COX-2 was detected by western blotting. The results showed that compared with the NT group, LPS significantly increased the protein levels of TNF-α (Figure 5B), IL-6 (Figure 5C), and COX-2 (Figure 5D,E), and that peiminine inhibited the production of these pro-inflammatory mediators (Figure 5B–E). However, the NT group and the NT + peiminine group were not different (Figure 5B,C). These results suggested that peiminine inhibited the inflammatory response induced by LPS in mMECs and did not cause an inflammatory response in normal mMECs.

### 2.5. Effect of Peiminine on the LPS-Induced Activation of AKT, NF-κB p65, ERK1/2, and p38 in mMECs

**Figure 6:**
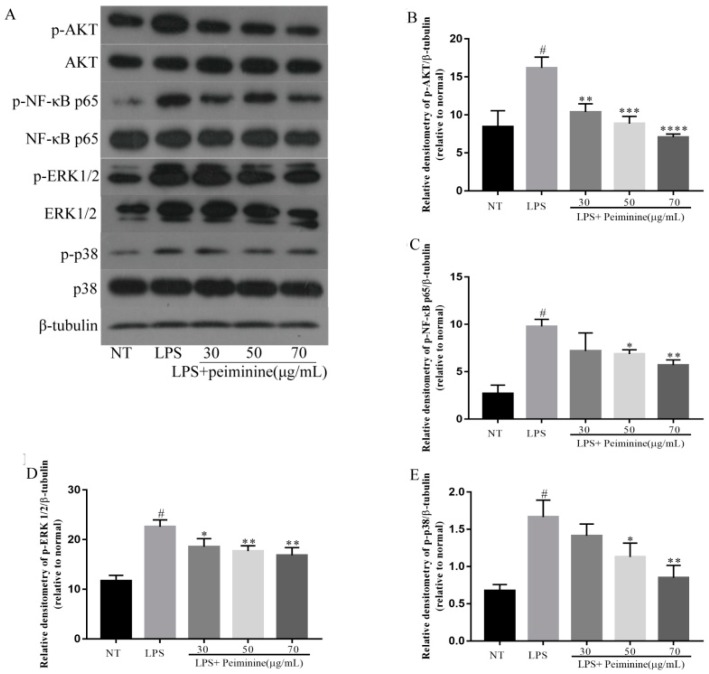
Peiminine inhibits the phosphorylation of the AKT, NF-κB p65, ERK1/2, and p38 signaling pathways in mMECs. The results for the protein levels of (**A**,**B**) p-AKT, (**A**,**C**) p-NF-κB p65, (**A**,**D**) p-ERK1/2, and (**A**,**E**) p-p38 were measured by western blotting. The data are presented as the mean ± SD (*n* = 6). The number sign (#) indicates a significant difference from the NT group at *p* < 0.0001. The tetrad asterisks (****) indicate *p* < 0.0001, the triple asterisks (***) indicate *p* < 0.0005, the double asterisks (**) indicate *p* < 0.01, and the single asterisk (*) indicates *p* < 0.05 vs. the LPS group.

In mice with LPS-induced mastitis, the authors found that peiminine inhibited the LPS-induced activation of AKT, NF-κB p65, ERK1/2, and p38 in the mammary tissues. To further demonstrate whether the effect of peiminine on inflammatory response was associated with these signaling pathways, the effect of peiminine on the phosphorylation of AKT, NF-κB p65, ERK1/2, and p38 was determined in the LPS-stimulated mMECs. The results showed that peiminine significantly suppressed the LPS-induced phosphorylation of AKT, NF-κB p65, ERK1/2, and p38 in a dose-dependent manner (Figure 6A–E).

## 3. Discussion

Peiminine is an alkaloid extracted from the traditional Chinese medicine Fritillaria that possesses a variety of biological properties [13,14,15]. A previous study confirmed that when the amount of peiminine in vivo reached 5 mg/kg, there were no toxic side effects [10]. In this study, an intraperitoneal administration of peiminine was performed twice, at 1 h before and 12 h after the LPS injection, and in vitro, the mMECs were pretreated with various concentrations of peiminine for 1 h and were then stimulated with LPS for 4 h. This study revealed that peiminine suppressed the pathologic changes to the mammary gland and inhibited MPO activity and production of pro-inflammatory mediators (TNF-α, IL-6, IL-1β, COX-2, and iNOS) by suppressing the AKT/NF-κB, ERK1/2, and p38 signaling pathways.

LPS-induced mastitis involves neutrophil infiltration as the main characteristic of the inflammatory response [16]. MPO is a distinguished enzyme in innate defense that is released by neutrophils, macrophages, and monocytes during phagocytosis into the extracellular environment, and MPO also takes part in various biological processes [17,18]. MPO is characterized by pro-oxidative and pro-inflammatory properties and is a feasible marker for a variety of inflammatory diseases, including acute lung inflammation [19] and acute intestinal inflammation [20,21]. In this experiment, MPO activity was significantly lower in the peiminine groups, which means that the neutrophil inflammatory infiltration decreased, indicating that peiminine had a certain therapeutic effect on mastitis. This may be related to the fact that peiminine restrained the excessive release of inflammatory cytokines induced by LPS. In addition, in order to evaluate the histological changes of the mouse mammary gland after LPS injection, the authors stained the mammary with hematoxylin and eosin. The results showed that LPS-induced hyperplasia of the glandular wall in the mammary tissues was significantly increased and showed a large number of inflammatory cells infiltrating, causing a serious inflammatory response. However, the peiminine groups displayed reduced inflammatory cells and a decreased neutrophil content. With increasing peiminine concentrations, the neutrophil content showed a significantly decreasing trend, especially at 5 mg/kg peiminine, at which point the status of the mammary gland was nearly normal. This result indicated that the different concentrations of peiminine effectively reduced the inflammatory lesions in the mammary tissue.

Pro-inflammatory cytokines, such as TNF-α, IL-6 and IL-1β, play an important role in inflammatory responses [22]. Among them, the production of TNF-α is very important for the synthesis of NO in the mammary tissues stimulated by LPS [23], and various types of inflammation, including mastitis, are shown to be involved [24]. Similarly, the release of IL-1β during LPS-induced inflammation causes damage to cells or tissues [25]. IL-6 is also an important pro-inflammatory factor that is considered to be the endogenous molecule that causes fever [26]. In this study, TNF-α, IL-6 and IL-1β levels were significantly increased in the LPS-induced mastitis model, and peiminine inhibited the production of TNF-α, IL-6 and IL-1β in a dose-dependent manner. This study’s results were consistent with those of Wang, who found that peiminine mitigated the pulmonary functional and structural impairment in chronic obstructive pulmonary disease (COPD) model rats and inhibited the inflammatory response [27]. Pro-inflammatory enzymes (COX-2 and iNOS) are induced during pathophysiological responses by inflammatory stimuli such as LPS, and their catalytic products promote the progression of the inflammatory response. Therefore, to investigate the mechanism of how peiminine attenuates LPS-induced mastitis in mice, the authors examined the effects of peiminine on LPS-stimulated mMECs. They found that peiminine also inhibited the secretion of inflammatory cytokines in LPS-induced mMECs, which was consistent with the results of the animal experiments. Thus, they determined the effect of peiminine on pro-inflammatory enzyme production. The results revealed that peiminine significantly decreased the LPS-induced production of COX-2 and iNOS. These results indicated that peiminine suppressed the LPS-induced inflammatory response in the mammary tissues. At the same time, Lim also confirmed the authors’ results regarding the effect of peiminine on DNCB-induced atopic dermatitis [28].

NF-κB is composed of p50 and p65 and is one of the most important regulatory factors for pro-inflammatory gene expression [29]. NF-κB plays a central role in the inflammatory response by controlling the expression of cytokines [30], such as TNF-α and IL-6 [31]. To confirm whether peiminine was involved in the anti-inflammatory response, the authors further investigated the impact of peiminine on the phosphorylation of NF-κB p65 and its upstream kinase AKT. The results revealed that LPS significantly induced the phosphorylation of NF-κB p65 and AKT, and that peiminine inhibited this effect in a dose-dependent manner. In 2015, Wu used RAW264.7 macrophages to confirm that peiminine inhibited the pro-inflammatory effect of LPS stimulation through the NF-κB pathway [32]. This result was similar to that of Bina Lee in 2015 in a study of the allergic inflammatory response induced by human mast cell-1 (HMC-1) cells mediated by peiminine [33]. Their results confirmed that the peiminine dose-dependent inhibition of LPS-induced secretion was similar to the TNF-α, IL-6 and IL-1β inflammatory cytokines, inhibiting the activation of NF-κB and the predominant degradation of IκB [33]. ERK1/2 and p38 mediate inflammation, and they are the important targets of anti-inflammation [34]. The authors also detected the effect of peiminine on the phosphorylation of ERK1/2 and p38. The results showed that LPS significantly enhanced the phosphorylation of ERK1/2 and p38, and that these effects were inhibited by an intraperitoneal administration of peiminine. In vitro, the AKT/NF-κB, ERK1/2, and p38 signaling pathways were also inhibited by peiminine, which was consistent with the results of the animal experiments. Previous studies have shown that peiminine can protect dopaminergic neurons from inflammation-induced cell death by inhibiting the ERK1/2 and NF-κB signaling pathways [35]. The above results indicated that the effect of peiminine on pro-inflammatory cytokines was mediated, at least in part, by inhibiting the AKT/NF-κB, ERK1/2, and p38 signaling pathways. 

**Figure 7:**
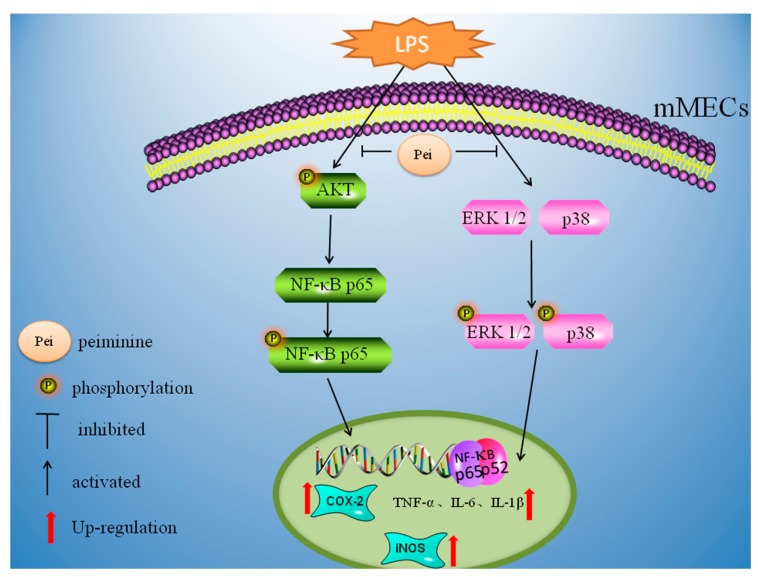
Peiminine inhibits LPS-induced inflammation by suppressing the phosphorylation of the AKT, NF-κB p65, ERK1/2, and p38 signaling pathways.

In conclusion, this study showed that peiminine had anti-inflammatory activity that depended on its ability to regulate the production of TNF-α, IL-1β, and IL-6 by suppressing the activation of the AKT/NF-κB, ERK1/2, and p38 signaling pathways. Moreover, the authors hypothesized that this was the mechanism by which peiminine decreased the production of inflammatory cytokines to provide an anti-inflammatory effect in a mouse LPS-induced mastitis model (Figure 7).

## 4. Materials and Methods

### 4.1. Animals

All the animal care and experimental procedures in the study were conducted in accordance with the guidelines established by the Jilin University Institutional Animal Care and Use Committee (approved on 27 February 2015, Protocol No. 2015047). BALB/c mice were purchased from the Center of Experimental Animals of the Baiqiuen Medical College of Jilin University (Jilin, China). Breeding triads, consisting of two females and one male, were randomly established and were supplied with food and water ad libitum during the experiments. The room temperature was kept at 25 ± 1 °C, and the water was sterilized by autoclaving. When pregnancy was confirmed, each female was housed individually.

### 4.2. Mouse Mastitis Model

Female and male mice were first housed together for 4–6 days to adapt them to the surroundings. Then, two female mice and one male mouse were randomly separated into each cage, which was supplied with sufficient water and forage material. Five to seven days after giving birth, the lactating females were isolated from their offspring, and they were randomly divided into five groups as follows: NT group (*n* = 6); LPS group (*n* = 6); and LPS + peiminine (1, 3, or 5 mg/kg) group (*n* = 6). The drug treatment group was pretreated by an intraperitoneal administration of peiminine (1, 3, or 5 mg/kg) (dissolved in Dimethyl sulfoxide (DMSO)) (>98% purity; Shanghai Yuanye Bio-Technology Co., Ltd., Shanghai, China). One hour later, the LPS-induced (dissolved in phosphate buffered solution (PBS)) (Sigma-Aldrich, St. Louis, MO, USA) mastitis model was established in the mice, with the exception of the NT group. The fourth pair of nipples and the surrounding area were sterilized with 75% ethanol. Then, the nipples were cut off at 1 mm from the peak in order to expose the milk ducts. Each teat duct was infused with 50 μL of 0.2 mg/mL LPS. After 12 h, the peiminine treatment group was injected with the same dose of different concentrations of peiminine. All the mice were sacrificed after 12 h, and the mammary tissues were harvested and frozen at −80 °C or were soaked in a 4% paraformaldehyde solution.

### 4.3. Cell Culture

The mMECs were purchased from the American Type Culture Collection (ATCC, ATCC^®^ CRL-3063™) and were cultured in dulbecco’s modified eagle medium (DMEM) (Gibco, Grand Island, NY 14072, USA) containing 10% fetal bovine serum (FBS) (Clark Bioscience, Richmond, VA, USA) at 37 °C in a humidified incubator with 5% CO_2_. Culture medium was changed every two days. When the cells grew to approximately 60–80%, the cells were passaged or stimulated.

### 4.4. Histopathologic Evaluation of Mammary Tissues

For histological analysis, the mammary tissues were fixed in fresh 4% formaldehyde solution for 24 h and were then dehydrated, transparent, dipped wax, and embedded in paraffin. Finally, 5-μm sections were cut and stained with hematoxylin-eosin. The tissue sections were observed under a light microscope to examine the mammary histopathology.

### 4.5. Tissue Homogenates and MPO Assay

One mammary tissue from each group was weighed and homogenized in hepesfreeacid (HEPES) at a ratio of 1:4 on ice. After it was fully grinded, the homogenate was transferred to a new suitable centrifuge tube and was then centrifuged at 13,000 rpm for 20 min. The supernatant was collected into a new centrifuge tube for ELISA, and the sediment was combined with 0.5% cetyltrimethylammonium chloride (CTAC) equal to HEPES and was centrifuged again at 13,000 rpm for 20 min in order to get rid of the remaining lipid. The supernatant was the MPO sample. Each MPO sample (75 μL) and substrate (75 μL), 3,3′,5,5′-Tetramethylbenzidine 3 mM (8798 μL), Resorcinol 6 mM (180 μL), and H_2_O_2_ 3% (2.5 μL) were added to a 96-well plate for 3–5 min. Finally, 100 μL of H_2_SO_4_ (2 M) was added to terminate the reaction. The absorbance peak (OD value) was detected at a wavelength of 450 nm by a microplate reader. The concentration of the analyte was based on the OD value.

### 4.6. Enzyme-Linked Immunosorbent Assay

The protein levels of TNF-α, IL-6, and IL-1β in the supernatant of the homogenate were evaluated with the corresponding ELISA kits according to the manufacturer’s instructions (Biolegend, San Diego, CA, USA). A total of 50 μL of diluted capture antibody solution was added to each well, and the plate was sealed and incubated overnight at 4 °C. The plates were then washed 4 times with wash buffer (0.05% Tween-20 in tris-buffered saline (TBS)) and were then blocked by adding 100 μL of assay diluent to each well. The plates were sealed and incubated at room temperature for 1 h with shaking on a plate shaker. All the subsequent incubations with shaking were performed similarly. The plates were washed 4 times, and 50 μL of the diluted standards and samples were added (at a 10-fold dilution) to the appropriate wells. The plates were sealed and incubated at room temperature for 2 h with shaking. The plates were then washed 4 times, and 50 μL of the diluted detection antibody solution was added to each well. The plates were sealed and incubated at room temperature for 1 h with shaking. Then, the plates were washed 4 times, and 50 μL of the diluted avidin- horseradish peroxidase (HRP) solution was added to each well. The plates were sealed and incubated at room temperature for 30 min with shaking. The plates were washed 4 times, soaking for 30 s to 1 min per wash. After adding 50 μL of 3,3′,5,5′-Tetramethylbenzidine (TMB) substrate solution to each well, the plates were incubated in the dark for 15–30 min or until the desired color developed. The reactions were then stopped with 50 μL of 2 M H_2_SO_4_, and the OD450 was measured. The TNF-α, IL-6, and IL-1β concentrations were determined using the standard curve generated from the known values.

### 4.7. CCK8 Assay

The effect of peiminine on cell viability was determined using the CCK8 assay. The mMECs were treated with peiminine, at concentrations ranging from 30 to 70 μg/mL, for 1 h and were then stimulated with LPS for 4 h. Subsequently, 10 μL of CCK8 (Saint-Bio, Shanghai, China) was added to each well. After 1 h, the absorbance (OD) was measured at 450 nm on a microplate reader.

### 4.8. Cell Culture Experimental Design

Mouse MECs were cultured in 60 mm × 15 mm cell culture dishes (Life Science, Oneonta, NY, USA) and were divided into groups as follows: NT; NT + peiminine 70 μg/mL; LPS; and LPS + peiminine (30, 50, or 70 μg/mL). When the mMECs reached 80% confluence, the serum-containing medium in the cell culture dishes was changed to serum-free DMEM for 3 h to reduce the mitogenic effects. After 3 h, the cells were pretreated with various concentrations of peiminine. One hour later, LPS was added. The cell supernatant or protein from the mMECs was extracted after 4 h.

### 4.9. Western Blot Analysis

The mammary tissues were lysed in lysis buffer (Beyotime, Shanghai, China). The protein concentrations were measured using the bicinchoninic acid (BCA) protein assay kit (Beyotime, Shanghai, China). A total of 30 μg protein was separated by 10% sodium dodecyl sulfate-polyacrylamide gel electrophoresis SDS-PAGE. The separated proteins were subsequently transferred onto a polyvinylidene difluoride (PVDF) membrane (Millipore, Darmstadt, Germany). After blocking with 5% nonfat milk for 2 h at room temperature, the membranes were incubated overnight at 4 °C with primary antibodies against COX-2 (1:1000), iNOS (1:2000) (Abcam, Cambridge, CA, USA), phospho-NF-κB p65 (1:1000), NF-κB p65 (1:1000), phospho-AKT (1:2000), AKT (1:2000), phospho-p38 (1:2000), p38 (1:1000), phospho-ERK1/2 (1:2000), ERK1/2 (1:2000) (Cell Signaling Technology, Danvers, MA, USA), and β-tubulin (1:2000) (Bosterbio, Pleasanton, CA, USA). Subsequently, the membrane was washed five times in 0.05% tris-buffered saline with Tween-20 (TBST, pH 7.4) for 10 min each time and was then incubated with an HRP-conjugated anti-mouse (1:3000) or anti-rabbit secondary antibody (1:3000) (Bosterbio) for 1 h on a shaker at room temperature. The membrane was again washed five times for 10 min each. Finally, an enhanced chemiluminescence detection kit (Beyotime, Shanghai, China) was used to visualize the immunoreactive proteins [36].

### 4.10. Data and Statistical Analysis

The images were produced using GraphPad prism software. The animals were randomly assigned to groups. In the mouse studies, the histological analysis was conducted in a blinded manner. Based on extensive experience with mouse models of LPS and the planned analytical framework, the authors estimated the number of mice per group required to detect the effects of interest at the *p* < 0.05 level of significance. The numbers of technical or biological replicates (independent experiments for individual mice for in vivo experiments) in each group are specified in the respective figure legends. All the data are presented as the means ± SD, as stated in the figure legends. A one-way ANOVA (general linear model) was applied for comparisons of more than two groups. The analyses were performed using GraphPad Prism 7.00 software (La Jolla, CA, USA).
